# Enhanced adaptive sliding mode control with termination parallelism cooperative optimization for torque ripple reduction in switched reluctance motor drives

**DOI:** 10.1038/s41598-025-96326-7

**Published:** 2025-05-09

**Authors:** G. Karthikeyan, T. Suresh Padmanabhan

**Affiliations:** 1 Department of Electrical and Electronics Engineering, Anjalai Ammal Mahalingam Engineering College Kovilvenni, Kovilvenni, Tamilnadu India; 2 Department of Electrical and Electronics Engineering, E.G.S. Pillay Engineering College, Nagapattinam, Tamilnadu India

**Keywords:** Adaptive sliding mode control (ASMC), Torque ripple reduction, Switched reluctance motor (SRM), Termination parallelism cooperative optimization (TPCO), Motor speed control, Electrical and electronic engineering, Mathematics and computing

## Abstract

Switched Reluctance Motor (SRM) technology has gained significant attention in various industries due to its reliability, cost-effectiveness, and efficiency. However, torque ripple remains a major issue, impacting the performance of SRM drives. This paper presents an enhanced Adaptive Sliding Mode Control (ASMC) strategy to reduce torque ripple in SRM drives. The proposed control scheme integrates an outer-loop ASMC controller for speed regulation and an inner-loop ASMC controller combined with a hysteresis controller for precise torque control, with the primary goal of minimizing ripple torque while maintaining the desired motor speed. The design process begins with the development of an 8/6 SRM model in MATLAB/Simulink, followed by the implementation of the control architecture. An advanced optimization technique, Termination Parallelism Cooperative Optimization (TPCO), is used to fine-tune system parameters and improve performance. The results demonstrate significant improvements in torque ripple reduction under different load conditions, achieving reductions of up to 80% under light load and 36.84% under heavy load conditions. These findings highlight the effectiveness of the proposed ASMC strategy in reducing torque ripple and improving efficiency, providing a promising solution for SRM-based drive systems.

## Introduction

Growing concern on Sustainable transportation has encouraged development of EVs in the world. SRM constitute as prospective electric motor technologies mainly because of their robust structure, high reliability, and low cost^1,2^. However, torque ripple is the main hindrance to their widespread use in EVs because it leads to unwanted acoustic noise and vibrations reducing the driving efficiency and passengers’ comfort^3,4^. The SRMs was determined mainly by the salient structure, as well as nonlinear magnetic characteristics. This problem does interfere not only with the fluidity of the car’s operations but also with the declining value or the popularity of the mechanical part and that ultimately leads to the increase in the maintenance cost and the reduced life expectancy of the motor. Thus, it is vital for SRM driven EV’s efficiency and enhanced efficiency to develop efficient control solutions to regulate torque ripple^5,6^.

Concerning torque ripple which is a common problem in SRMs, different techniques such as advance control techniques and mechanical changes have been applied^7^. Among the described methods, control techniques can be considered effective and suitable mainly because it do not involve drastic changes to the motor’s design. Such strategies frequently revolve round increasing the present profile, utilizing higher modulation techniques, or utilizing crisp control frameworks to reliably alter the nature of motor performance depending on operational^8,9^. Voltage monitoring is a popular method which seeks to look at phase currents so as to provide a better input for torque. The strategy of this system is effective and it responds quickly by bouncing off the torque ripple. The other efficient technique is Pulse Width Modulation (PWM) system, which regulates the voltage control of power electronics and torque ripple. The model predictive control (MPC) technique and adaptive controller schemes have been shown to under potential for dropping the ripple of torque by adjusting the control signals recursively based on motor parameter variations and/or load disturbances^10^.

The integration of these control systems with current sensors and real-time data processing capabilities improves their efficacy^11^. Sensor less control systems, which estimate motor voltage and current, have also gained appeal as a low-cost alternative to traditional sensor-based approaches. Researchers and engineers can significantly improve the operational smoothness and overall efficiency of SRM-powered EVs by developing new control structures, making more realistic and desirable in the competitive automobile market^12^.

The authors use ASMC to enhance system robustness, but the primary control method remains hysteresis control. Model Predictive Control can reduce harmonic content in current and torque by optimizing switching states and voltage vectors^13^, thereby improving system efficiency and performance. In contrast, hysteresis control tends to result in higher harmonic content. Additionally^14^, proposes a different robustness approach, using a sliding mode observer to estimate lumped disturbances in the predictive model parameters^15^. model-free schemes, online parameter identification schemes, and observer-based disturbance compensation strategies, which differ significantly from the robustness principles of SMC.

## Related work

A novel technique of Direct Torque Control (DTC) utilizing SMC technology was presented in^16^ that reduced torque ripples in an SRM. The SMC treatment changes the reference current value and thus addresses low-frequency fluctuations in torque production and ensures constant motor speed. It was concluded that when implemented continuously, SMC outperformed PI controller notably in torque ripple suppression, torque nonlinear feature tuning, and the system’s ability to respond to variable changes. A fuzzy logic controller (FLC) in DTC was used to decrease the torque ripple of an SRM motor in EVs^17^. Along with a set of 49 rules, the FLC takes in speed and speed error inputs. The drive becomes less sensitive to changes in parameters and compensates for torque characteristics that were not linear, as demonstrated by the results, which indicated that FLC decreased motor torque ripple more efficiently than PI controllers.

For EV propulsion systems, a hybrid design approach for segmented rotor double-sided axial flux SRMs (DSAFSRM) was described in^18^. Without sacrificing efficiency, torque ripple was reduced by a two-step optimization process. Regarding efficiency, power density, torque ripple, and average torque, the optimized DSAFSRM performs better than a double-sided radial flux SRM. The average torque of the improved DSAFSRM was 26% higher. A novel, improved control strategy for Minimum Torque Ripple Point Tracking (MTRPT) of a 4-stage SRM having 6/8 poles was presented in^19^. Using a table of search results, the approach regulated turn-on and transmission angles while changing the present state in the start-up mode. The approach also featured a lookup table to limit the turn-off angles and an adjustable turn-on angle that was proportionate to the applied speed.

An EV’s photovoltaic module, battery, and SRM energy flow might be regulated by a tri-port inverter, which was based on research^20^. Closed-loop velocity management was accomplished using proportionate and integral control, fortunately, torque sharing function (TSF) was used in the indirect rotation control technique. In motor drive sector, hysteresis flow controllers were employed to create gate pulses, which led to reduced loss of copper and enhanced torque ripple reductions. The designs of DC-link capacitors in electric vehicle traction uses optimal pulse patterns (OPPs) to reduce the fluctuations in voltage and battery density examined by^21^. Energy consumption was necessary to save the power. A 250 kW test facility was used to experimentally confirm a 20% decrease in DC-link capacitance volume.

This paper examined reference torque neural network (RTNN) to modify the default torque to reduce the torque fluctuation. RTNN was a single-output network^22^. The torque fluctuation and rotor position have a periodic connection, and the rotor angle was the primary component of the explicit function in RTNN. The experimental outcome demonstrated how accurately the angle was measured^23^. proposed a Direct Instantaneous Torque Control (DITC) based on an adaptive turn-on angle technique to enhance the torque ripple of SRM in EVs. When it was in the minimal inductance region of the incoming phase, the control scheme follows the torque by using two operational modes that change the outgoing and incoming phase voltages. That was due to the closed-loop regulator increasing the motor efficiency as it was capable of varying the turn-on angle.

This paper investigated permanent magnet synchronous motors (PMSMs) with torque ripple for reducing the injecting harmonics^24^. The suggested approach improves the performance of motor and torque ripple reduction, for enhancing the productivity of electric vehicles and aerospace consumptions. Electric vehicles could reduce energy waste while preserving performance restrictions by using the permanent magnet-assisted synchronous reluctance (PMaSynR) motor examined by^25^. The optimized design reduces energy loss to 11.7% while driving vehicles, with a 4.7% torque peak reduction.

Based on the MPC-based algorithm with model-positioning, research^26^ offered application-oriented management of the SRM drive for LEVs. By employing rotor position signals for sector division, the technique was associated with low computational load and optimum voltage vectors. To extend the distance per charge for EVs and enhance efficiency in recovering kinetic energy in regenerative braking mode. 1.25 kW, 12/8, three-phase switched reluctance motor was adapted to experiment with the proposed method. In this a multilayer converter (MLC) is used to enhance torque anomaly control approach by direct instantaneous torque control (DITC) system to decrease the ripple of torque in SRMs for electric vehicles^27^. The experimental outcome demonstrated that suggested approach was efficient and reduces torque ripple.

An improved Direct Torque Control (DTC) method for Dual Star Induction Motors (DSIM) using the Grey Wolf Optimization (GWO) algorithm was proposed to reduce torque ripple, enhance response time, and improve speed control. The GWO-PID controller, tested through MATLAB simulations, showed better performance compared to the traditional PID-DTC method. However, the study did not evaluate performance under varying load conditions and different speed characteristics, making it unclear how well the controller adapts to dynamic changes^28^. The GWO-MFTSMC method improves efficiency, but it still faces challenges in further reducing the response time under dynamic load variation^29^.

The proposed ASMC strategy offers significant advantages, including effective reduction of torque ripple, improved efficiency, and precise speed control across various load conditions. It provides better performance compared to traditional control methods, ensuring smoother operation and enhanced system reliability. This method holds great potential for further advancements in SRM drive systems and broader applications in different industries.

## Methodology

**Fig. 1:**
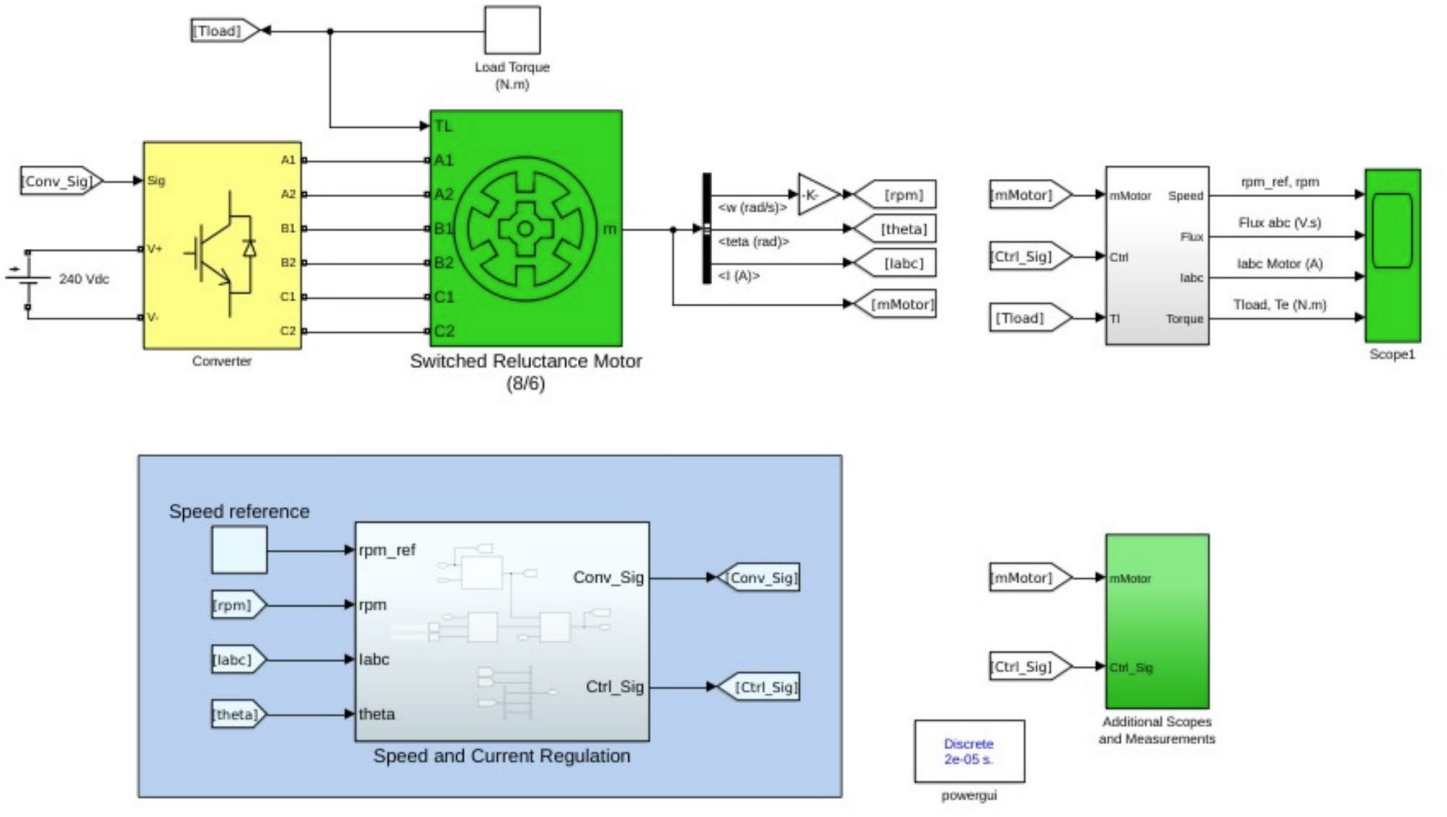
Simulation of SRM Drive.

**Fig. 2:**
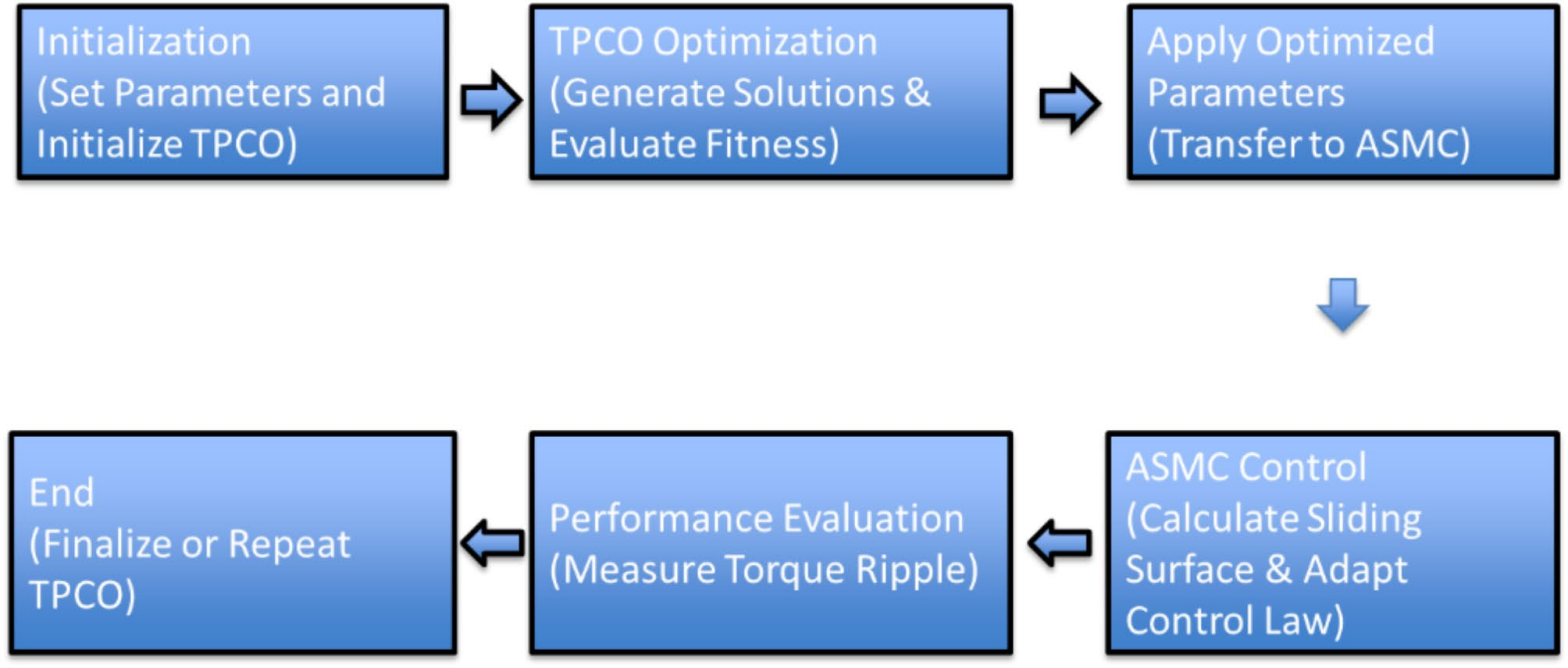
Workflow of TPCO-ASMC.

Research begins with designing an 8/6 Switched Reluctance Motor (SRM) using MATLAB\Simulink to model its electrical and mechanical characteristics. It implements an Outer Loop Adaptive Sliding Mode Control (ASMC) for speed regulation and an Inner Loop ASMC with a hysteresis controller for torque control. Optimization using TPCO fine-tunes controller parameters in the MATLAB\Simulink environment, ensuring optimized performance and robust control (Fig. 1). TPCO optimizes the control parameters to achieve minimal torque ripple, while ASMC provides robustness against disturbances and parameter variations. Figure 2 illustrates the Workflow of TPCO-ASMC.

### Design of switched reluctance motor (SRM)

The 8/6 SRM motor control system regulates speed using a closed-loop mechanism with real-time feedback. The converter module transforms 240 V into controlled signals to power the stator windings, enabling smooth rotation. Key parameters like speed, flux linkage, and phase currents are measured for performance evaluation. The 8/6 SRM motor control system regulates speed using a closed-loop mechanism with real-time feedback. The converter module transforms 240 V into controlled signals to power the stator windings, enabling smooth rotation. Key parameters like speed, flux linkage, and phase currents are measured for performance evaluation. This model enhances torque ripple reduction and ensures better speed regulation for improved efficiency and stability.

**Fig. 3:**
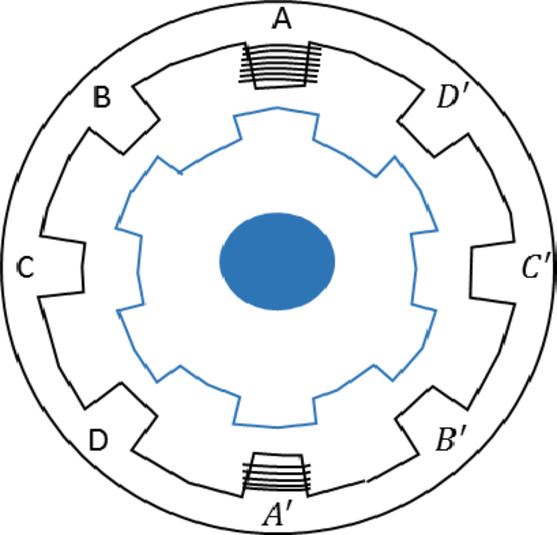
8/6 Structure of SRM’s.

An 8/6 SRM’s cross-section is seen in Fig. 3. This step involves modelling the motor to accurately represent its electrical and mechanical characteristics. This motor has a basic construction and is doubly salient synchronous. The rotor lacks coils and magnets, but the stator has concentrated field windings surrounding each tooth.

With the least amount of magnetic resistance, the electromagnetic system tends to reach a stable equilibrium position, which is the basis for the SRM’s operation. One of the stator windings is excited, and the distance between the rotor and stator poles that are energized determines the amount of torque that is generated. The motor spins when the stator winding is excited about the rotor position. The following voltage is present at the motor phases, using Faraday’s Law guidelines:

1 $$\:{V}_{j}={R}_{s}{i}_{j}+\frac{d{\lambda\:}_{j}(\theta\:,{i}_{j})}{dt\:}$$

Where$\:{V}_{j}$, $\:{i}_{j}$, $\:{\lambda\:}_{j}$, and $\:{R}_{s}$ stand for the corresponding $\:i$th one phase resistance, phase voltage, flux linkage, and phase current. Offered by:

2 $$\:{\lambda\:}_{j}\left(\theta\:,{i}_{j}\right)={L}_{j}(\theta\:,{i}_{j}){i}_{j}$$

Where the rotor’s location is indicated by $\:\theta\:$ and $\:{L}_{j}$ is the $\:i$th phase inductance. The rate at which the flux-linkage changes at a constant pace is:

3 $$\:\frac{d{\lambda\:}_{j}(\theta\:,{i}_{j})}{dt}=\frac{\partial\:{\lambda\:}_{j}}{{\partial\:i}_{j}}\frac{{di}_{j}}{dt}+\frac{\partial\:{\lambda\:}_{j}}{\partial\:\theta\:}{\omega\:}_{r}={l}_{j}\left({i}_{j},\theta\:\right)\frac{{di}_{j}}{dt}+{e}_{j}$$

In which the progressive inductance is denoted by $\:{l}_{j}$ and the back-emf by $\:{e}_{j}$. Torque properties of the 8/6, four-phase SRM utilized in this experiment. The measured flux linkage data may be used to calculate the progressive inductance and back-EMF properties of the SRM using numerical techniques.

### Control architecture design

**Fig. 4:**
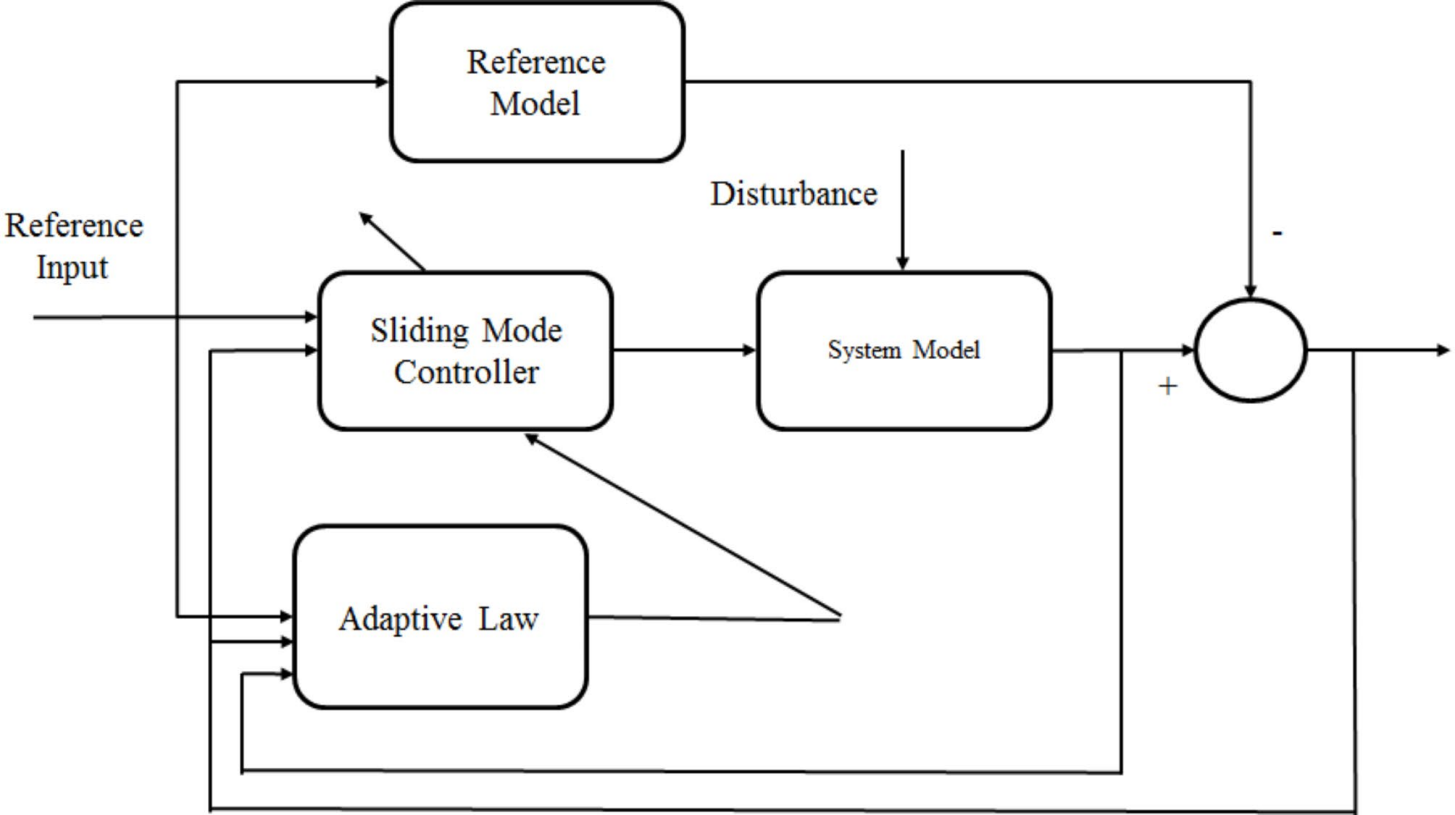
ASMC controller.

The control architecture features an Outer Loop ASMC Controller for speed regulation, optimizing ASMC. ASMC controller design is illustrated in Fig. 4. The Inner Loop ASMC Controller with Hysteresis Controller minimizes torque ripple using advanced optimization techniques like TPCO, ensuring robust performance in SRM drives for electric vehicles.

### Outer loop ASMC controller

The torque ripple arises from the nonlinear torque current-angle (S - j - θ) features of SRM since the torque-producing process is discontinuous. The most noticeable torque ripple occurs at commutation instants when torque production shifts from one active state to another due to SRM’s doubly salient structure. A motor’s inductance rising with increasing shaft angle results in positive torque also known as motoring torque. Similarly, sending current to the SRM winding as the motor’s inductance decreases results in negative torque (braking). To lessen torque ripple in driving mode, avoid going into negative torque districts. To reduce torque ripples in SRM drives, adjust current, and alter on and off angles. The hysteresis controller for each step of the present control loop is identical.

Determining asymptotically stable surfaces is the objective of the SMC technique. Trajectories of the system congregate on these surfaces and slide across until it cross, obtaining the origin. However, selecting appropriate heuristic sliding settings is crucial. A multi-objective method is utilized to identify the optimal settings, eliminating the need for experimentation. SRM magnetic saturation was taken into consideration when optimizing ASMC environments and turning on and off angles ($\:{\theta\:}_{on}$and $\:{\theta\:}_{off}$) using TPCO. To optimize these factors, aim to decrease the Integral Squared Error (ISE) of ripples in speed and torque. Equations 4 and 5, respectively, are used to determine the ISE of torque and speed.

4 $$\:IS{E}_{Speed}=\int\:{({\omega\:}_{ref}-\omega\:)}^{2}ds\:$$

5 $$\:ISE\_Torque=\int\:{\left({S}_{ref}-{S}_{f}\right)}^{2}ds$$

Therefore, these are the two goals as stated:

6 $$\:{e}_{1}=\text{m}\text{i}\text{n}(ISE\_Speed)$$

7 $$\:{e}_{2}=\text{m}\text{i}\text{n}(ISE\_Torque)$$

Here is how to calculate the torque ripple coefficient:

8 $$\:{S}_{j}=\frac{{S}_{f\_max}-{S}_{f\_min}}{{S}_{f\_mean}}$$

Where the values of the entire torque are represented by the terms $\:{S}_{f\_max}$, $\:{S}_{f\_min}$, and $\:{S}_{f\_mean}$, respectively.

### Inner loop ASMC controller with hysteresis controller

As soon as the system arrives at the surface that slides and stays there, SMC is more resistant to changes in internal parameters and external disturbances than other nonlinear control techniques. When an interrupted function is utilized or a large value is assigned to the sliding gain, the chattering phenomenon occurs. Thus, a technique that lessens chattering has been developed for estimating the unknown assumptions of a lump without utilizing $\:sgn(.)$. Use the following mechanical equation when designing a speed controller:

9 $$\:\dot{\omega\:}=\left(\frac{1}{I}\right)\left[{S}_{f}\left(\theta\:,{j}_{l}\right)-{S}_{K}-{C}_{\omega\:}\right]$$

Considering uncertainty as

10 $$\:\frac{d\omega\:}{ds}=\left(b+\varDelta\:b\right)\omega\:+\left(a+\varDelta\:a\right)\left({S}_{f}-{S}_{K}\right)$$

Where $\:b=-\frac{C}{I}anda=\frac{1}{I}$ The speed error state variable, defined as $\:f={\omega\:}^{\text{*}}-\omega\:$ changing surface, may be defined as follows:

11 $$\:{T}_{c}=\sigma\:f+\dot{f}$$

and choosing the Lyapunov function as follows:

12 $$\:{U}_{1}=\frac{1}{2}{T}_{c}^{2}$$

13 $$\:{\dot{U}}_{1}={T}_{c}{\dot{T}}_{c}={T}_{c}\left[-\left(\sigma\:+b\right)\dot{\omega\:}-av+{a\dot{S}}_{K}-a\left(\stackrel{\sim}{O}+\widehat{O}\right)\right]$$

$$\:O\left(s\right)=1/a\left[\varDelta\:b.\omega\:+\varDelta\:a\left({\dot{S}}_{f}-{\dot{S}}_{K}\right)\right]$$

14 $$\:\stackrel{\sim}{O}\left(s\right)=O\left(s\right)-\widehat{O}\left(s\right)$$

The estimated value of the packed uncertainty is denoted by $\:\widehat{P}\left(t\right)$, and the estimated variance between the real value $\:P\left(t\right)$ and the expected value of the aggregated uncertainty is represented by $\:\stackrel{\sim}{P}\left(t\right)$. Consequently, the newly proposed candidate role is

15 $$\:{U}_{2}=0.5\left({T}_{c}^{2}+\left(\frac{1}{\rho\:}\right){\stackrel{\sim}{O}}^{2}\right)$$

16 $$\:{\dot{U}}_{2}={T}_{c}\left[-\left(\sigma\:+b\right)\dot{\omega\:}-a\left(v+\dot{O}\right)+{a\dot{S}}_{K}+{L}_{1}{T}_{c}\right]-\stackrel{\sim}{O}\left(\frac{1}{\rho\:}\widehat{O}+{aT}_{c}\right)-{L}_{1}{T}_{c}^{2}$$

Assuming control input $\:v$ is selected as:

17 $$\:v=\frac{1}{a}\left[-\left(\sigma\:+b\right)\dot{\omega\:}-a\widehat{O}+a{\dot{S}}_{K}+{L}_{1}{T}_{c}\right]$$

18 $$\:{\dot{U}}_{2}=-{L}_{1}{T}_{c}^{2}-\stackrel{\sim}{O}\left(\frac{1}{\rho\:}\widehat{O}+{aT}_{c}\right)$$

19 $$\:\widehat{O}=-\rho\:a{T}_{c}$$

20 $$\:{\dot{U}}_{2}=-{L}_{1}{T}_{c}^{2}\le\:0$$

Applying the Lyapunov equilibrium theorem, after using the adaptation law, it is feasible to prove that the speed error gets to zero at infinite time.

### Optimization process

Consequently, Termination Parallelism Cooperative Optimization (TPCO), an advanced optimization technique designed to enhance the functionality of the control system, aims to fine-tune parameters to yield optimal outcomes. In contrast, TPCO is computationally superior to conventional optimization methods since it quickly and accurately identifies the best solution by implementing cooperation and parallelism.

### Termination parallelism cooperative optimization (TPCO)

The same case applies to the SRM control design where TPCO is employed to fine-tune the parameters of the ASMC controllers to help in achieving low ripple torque in the motor to enhance the necessary motor speed for efficient electric vehicle use. Based on the research, the following parameters are defined: Generation number, population size, mutation rate, crossover rate, and optimization variable for the ASMC strategy. These parameters are crucial to achieving high control efficiency and reduced torque ripple in SRM drives.

Fitness Evaluation: A fitness function, E(W), is used to evaluate each unique $\:{W}^{\left(i\right)}$ in the population. It determines how effectively the SRM configuration represented by W^((i)) reduces torque ripple and satisfies operational restrictions.

21 $$\:Fitnes{s}_{i}=E\left({W}^{\left(i\right)}\right)$$

This function includes torque ripple estimations, together with other constraints like current and voltage constraints.

Update Best Solution: Keep one or more track of the best option that has been found so far in terms of the fitness evaluations during the optimizing process,

22 $$\:{W}^{best}=\text{arg}\underset{i}{\text{min}}Fitnes{s}_{i}$$

This makes that approach preserves the best possible parameters found so far for the current generation.

Check Termination Criteria: Decide whether the process should be stopped or not based on some pre-defined criteria, which may include the target level of torque rippling or the maximum number of generations possible,

23 $$\:Terminate=criteriaMet\left(\right)$$

These can be employed to check whether further iterations are not likely to cause more significant leaps or when the fitness value has been fitted to the right Nash equilibrium.

The value of $\:\alpha\:$ represents a random crossover rate, which affects the quantity of genetic information passed between parents and typically ranges from 0 to 1.

Mutation: To investigate new parts of the response space while preventing premature convergence, and provide random modifications to offspring,

24 $$\:{w}_{i}^{\left(d\right)}={w}_{j}^{\left(d\right)}+\delta\:.\:\left({W}_{j}^{max}-{W}_{j}^{min}\right). \:q$$

In the above equation, $\:\delta\:$ denotes the mutation’s rate, and $\:q$ is a random number from − 1 to 1. This randomized technique enables the finding of possibly better options outside of the current local optimal condition.

Constraint Application: Confirm that offspring characteristics $\:{W}^{\left(d\right)}$ adhere to functional constraints such as current and voltage restrictions.

25 $$\:{W}^{\left(d\right)}=ApplyConstraints\:{W}^{\left(d\right)}$$

This phase is crucial for the improvement process yields solutions that are practical and feasible for practical implementation.

Torque Ripple Calculation: Analyse every offspring’s efficiency by measuring their torque ripple,

26 $$\:Torque\:Rippl{e}^{\left(d\right)}=CalculateTorqueRipple\left({W}^{\left(d\right)}\right)$$

The efficiency and operational stability of SRMs are directly impacted by torque ripple, which is a crucial performance indicator.

Population Update: To construct the next generation, replace the least fit members of the present population with the newly formed children.

27 $$\:Populatio{n}^{\left(next\right)}=\{{W}^{\left(d1\right)},\:{W}^{\left(d2\right)},\:\dots\:\}\:$$

The population will always get better because of this evolutionary process, leading to optimum or almost perfect solutions.

Display Best Fitness: To keep tabs on the advancement of the optimization process, track and present the current generation’s greatest fitness value,

28 $$\:BestFitness=\underset{i}{\text{min}}Fitnes{s}_{i}$$

When to break the optimization loop is assisted by this measure, which also sheds light on the standard of solutions being produced.

Parent Selection: Parents are chosen for crossover based on their fitness values, with the fitter parents getting priority,

29 $$\:Prob\left(i\right)=\frac{Fitnes{s}_{i}}{{}_{l=1}{}^{o}Fitnes{s}_{l}}$$

Using a stochastic selection procedure, beneficial features may be maintained and propagated by boosting the possibility that higher fitness will be selected as a parent.

Crossover: Combining the characteristics of chosen parents to produce new children

30 $$\:{W}^{\left(d1\right)}=\alpha\:{W}^{\left(o1\right)}+\left(1-\alpha\:\right){W}^{\left(o2\right)}$$

31 $$\:{W}^{\left(d2\right)}=\alpha\:{W}^{\left(o2\right)}+\left(1-\alpha\:\right){W}^{\left(o1\right)}$$

**Fig. 5:**
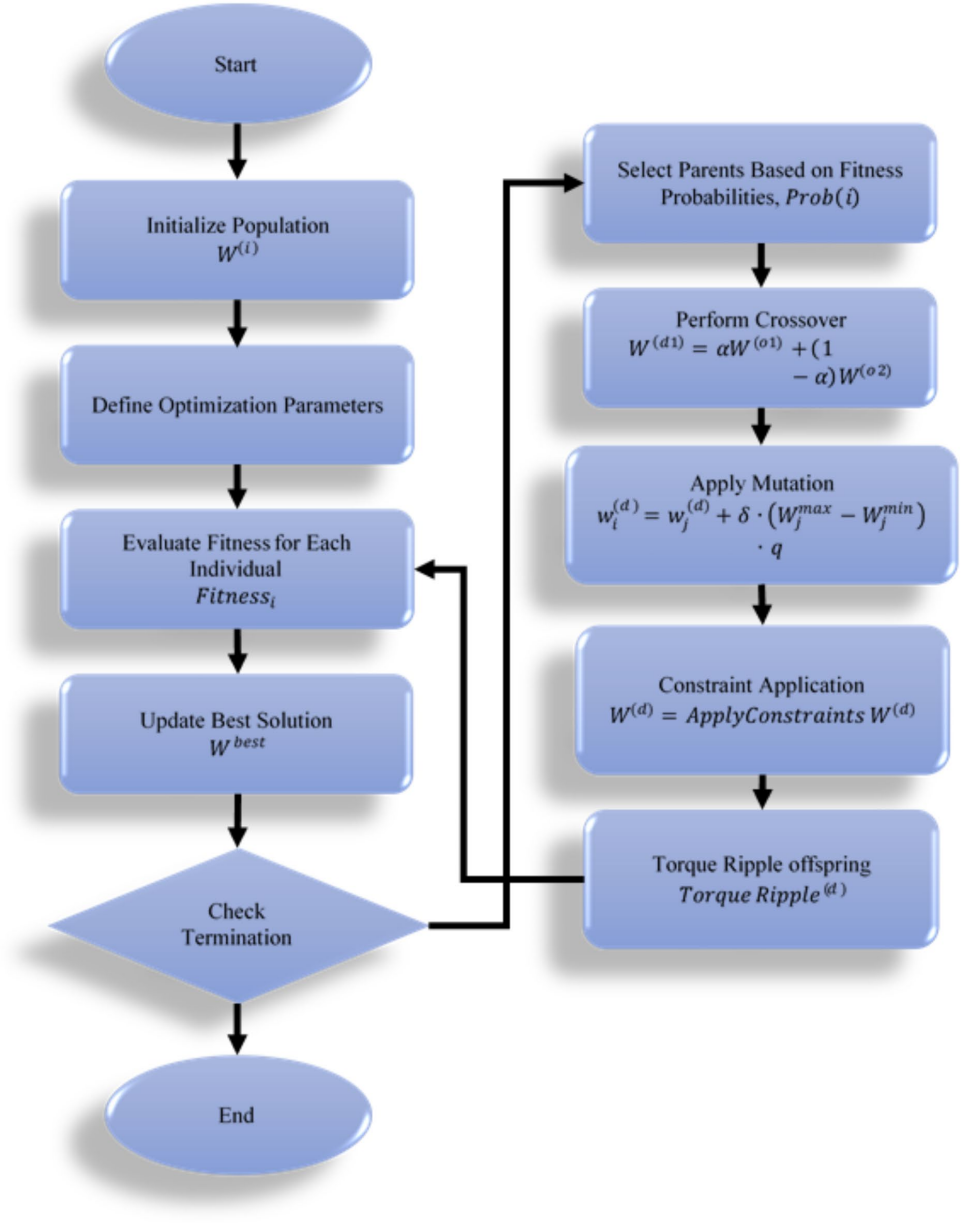
Flow chart of TPCO.

The flowchart illustrates the optimization process for torque ripple reduction using Termination Parallelism Cooperative Optimization (TPCO) is shown in Fig. 5. It begins with population initialization, followed by fitness evaluation, selection, crossover, mutation, and constraint application to refine solutions iteratively. The process continues until the termination criteria are met, ensuring optimal torque ripple minimization.

## Results

This section presents the results of simulating basic ASMC to illustrate the effectiveness of the latter. MATLAB/SIMULINK software is used to set up simulations for a four-phase, four-kw SRM. Magnetic saturation is taken into consideration in the model. Initial trial and error is used to determine the ASMC parameters; TPCO is then utilized for obtaining these parameters. SRM parameters are shown in Table 1. Table 2 summarizes the parameters utilized in the TPCO simulations. The effectiveness of the suggested optimization (TPCO) was analyzed by applying a set of parameters including Torque Ripple (Nm). Compared with existing optimization is “Harris Hawk’s Optimization (HHO)”^30^.

**Table 1:** Essential parameters of the 8/6 SRM.

Parameter	Value
SR Motor Inertia	0.008 Nm·s²
Rated Power	675 kW
System Inertia	0.04 Nm·s²
Rotor Poles	6
System Friction Coefficient	0.009 Nm·s
Stator Poles	8
Phase Resistance	0.09 Ω
Min. Inductance	10 mH
Rated Phase Current	50 As
Rated Voltage	240 V / phase

**Table 2:** TPCO parameters and constraints for ASMC control optimization.

Category	Parameter	Value	Description
**TPCO Parameters**	Num Generations	100	Number of generations for the optimization process.
	Population Size	50	Population size in the optimization algorithm.
	Mutation Rate	0.1	Mutation rate for the genetic algorithm.
	Crossover Rate	0.8	Crossover rate for the genetic algorithm.
**Optimization Problem Boundaries**	Lower Bound	[0.1, 0.1, 0.1]	Lower bounds for the optimization variables.
	Upper Bound	[10, 10, 10]	Upper bounds for the optimization variables.
**Additional Parameters for Control**	Sliding Surface Gain	0.5	Sliding surface gain for the control algorithm.
	Boundary Layer Thickness	0.3	Boundary layer thickness in the control algorithm.
	Hysteresis Bandwidth	1.2	Hysteresis bandwidth in the control algorithm.
	Inductance Values	[0.2, 0.3, 0.25]	Inductance values for each phase of the SRM.
	Resistance Values	[1, 1.2, 0.8]	Resistance values for each phase of the SRM.
**Optimization Constraints**	Max Voltage	100	Maximum allowable voltage.
	Max Current	50	Maximum allowable current.

**Fig. 6:**
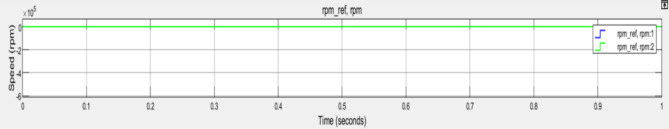
Performance of Speed response.

The x axis indicates the time, ranges from $\:0$ to $\:1$ second and y axis indicate the speed (rpm). Figure 6 represents the suggested methods performance Speed. An analysis of the behavior or performance of a system that depends on signals might benefit from the visual comparison of two reference speeds during a certain time period. It demonstrates how controller response to reference speed, in terms of their performance and stability characteristics.

**Fig. 7:**
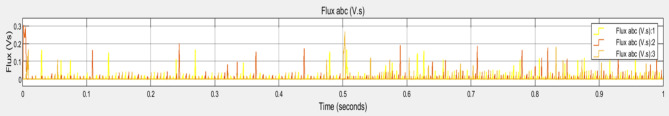
Graphical representation of Flux.

The flux values have periodic behaviour with an increasing amplitude and frequency in time. Figure 7 illustrates the graphical representation of flux. Where, x plane indicates time, ranges from $\:0$ to $\:1$ second and y plane indicates flux, spanning from $\:0$ to $\:0.3$ Wb. It demonstrates three distinct flux measurements. The significance of magnetic systems constitute the behaviour of flux over time. It helps to identify the performance or stability of the system.

**Fig. 8:**
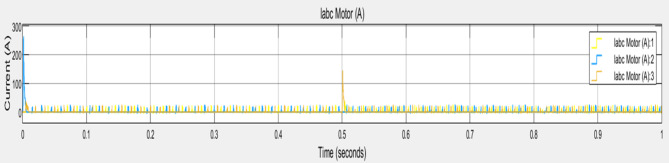
Graphical representation of Current peaks.

The x axis depicts the time in seconds, ranges from $\:0$ to $\:1\:$seconds and y axis indicates the current in Amperes, ranging from 0 to 300 A. Figure 8 represents the graphical representation of current peaks. Where yellow line illustrates the labc motor (A)1, blue line depicts the labc motor (A)2 and orange line represents the labc motor (A)3. It helps to identify the performance and stability of the motor phases like initial current spike, periodic oscillations and any anomalies.

**Fig. 9:**
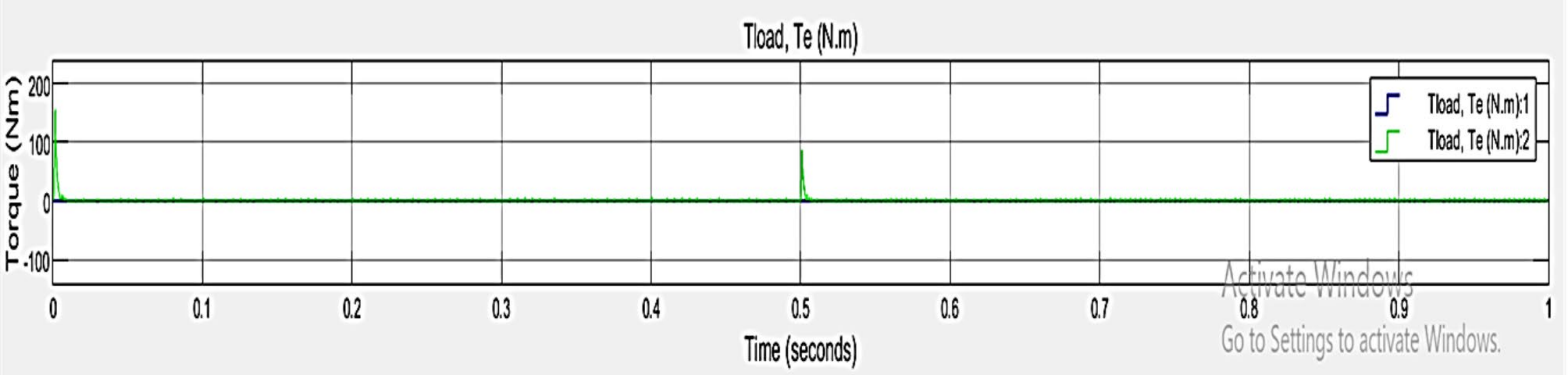
Graphical Representation of Torque dynamics.

Torque dynamics graphical representation, illustrated in Fig. 9. Where, x axis ranges from 0 to 1 s and y axis ranges from $\:-100\:$to $\:200\:Nm$ load torque. The green plot stars with 0 Nm at the beginning, after rises quickly to around 150 Nm and shows oscillations. At about 0.5 s, the green plot spikes and increases slightly before decreasing but oscillating around$\:90\:Nm$. It shows the difference and how systems react in different times at varying conditions.

### Case 1: Load – 0 NM, ripple torque (NM) = 0:6

**Fig. 10:**
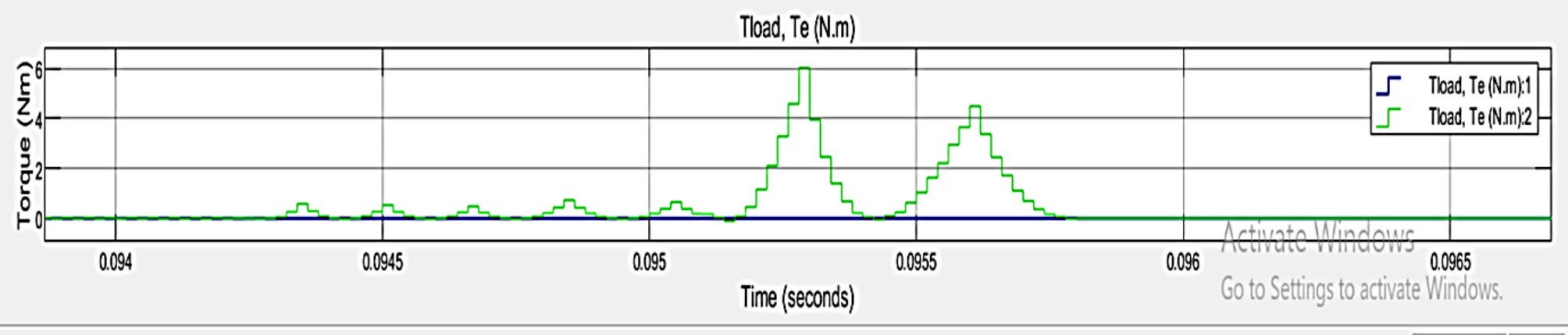
Performance Metrics of TPCO – Case 1.

The x axis indicates the time in seconds and y indicates the torque (NM). It shows torque over a very short period, from roughly 0.094 to 0.0965 s. Figure 10 represents the performance metrics of TPCO – Case 1. The green line generates more prominent peaks than the blue line. The blue line labeled the Tload, Te (N.m)1 and a green line labeled the Tload, Te (N.m) 2.

### Case 2: Load – 50 NM, ripple torque (NM) = 35:65

**Fig. 11:**
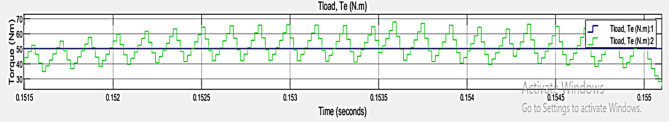
Performance Metrics of TPCO – Case 2.

The performance metrics of TPCO – Case 2, represented in Fig. 11. It shows torque in Newton-meters (N.m) over a very short period, from roughly 0.1515 to 0.155 s. The blue line labeled the Tload, Te (N.m)1 and a green line labeled the Tload, Te (N.m) 2. The flat portion of the blue line illustrates the torque steady state. The green line shows regular peaks and fluctuations in torque. It helps to analyze the performance of torque behavior in load 50 NM.

### Speed – 2000 rpm

**Fig. 12:**
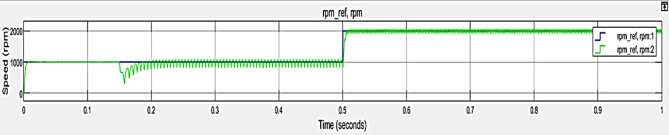
Performance Metrics of Case 2 -TPCO Speed.

The x plane represents time, ranging from 0 to 1 s, while the y plane represents the speed, ranging from 0 to 2000 rpm. Figure 12 depicts the Performance Metrics of Case 2 -TPCO Speed. The green line demonstrates several oscillations at the 1000 rpm level which shows unstable behavior or system noise. The blue line illustrates a steady transition. It shows how different control methods perform when regulating system speed during time periods.

### Case 3: Load – 100 NM, ripple torque (NM) = 60:120

**Fig. 13:**
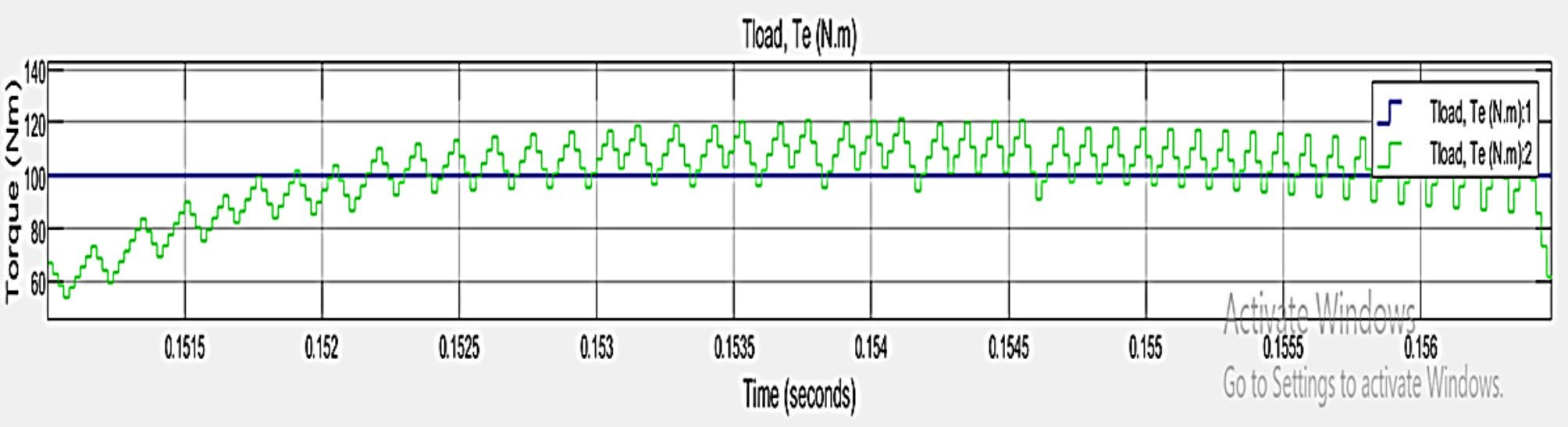
Performance Metrics of TPCO – case 3.

The x axis indicates the time in seconds and y indicates the torque (NM). Figure 13 represents the performance metrics of TPCO – Case 3. It shows torque (Tload, Te) over a brief time span, around 0.1515 to 0.1565 s. Tha blue line, indicates the Tload, Te (N.m) 1, which remains flat and stable, and a green line indicates the Tload, Te (N.m) 2, which exhibits regular peaks and troughs, signifying fluctuating torque. This visualization likely pertains to analyze torque behavior in mechanical or electrical systems, highlighting the contrast between stable and variable torque in load 100 NM.

### Speed – 2500 rpm

**Fig. 14:**
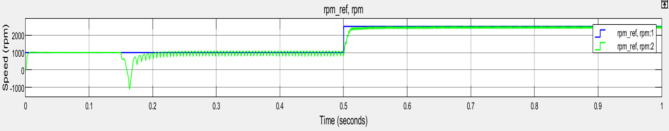
Performance Metrics of Case 3 -TPCO Speed.

The x plane represents time, ranging from 0 to 1 s, while the y plane represents the speed, ranging from $\:-1000$ to $\:2000$ rpm. Figure 14 depicts the Performance Metrics of Case 3 -TPCO Speed. It helps to demonstrate how two different speed control mechanisms operate regarding stability and performance when adjusting and maintaining speed duration. Data from the green line implies that rpm2 achieves speed control with less stability when compared to rpm1.

### Case 4: Load – 200 NM, ripple torque (NM) = 170:290

**Fig. 15:**
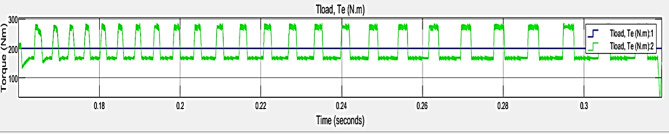
Performance Metrics of TPCO - case 4.

The performance metrics of TPCO – Case 4, depicted in Fig. 15. The x plane depicts the torque over a duration of 100 to 300Nm and y plane depicts the Time (sec). Where, the blue line indicates the Tload, Te (N.m) 1 it was stable, and a green line indicates the Tload, Te (N.m) 2 has periodic peaks and indicating torque fluctuations. It helps to identify how torque behaves in load 200 NM.

Table 3 compares the optimization results with existing methods for minimizing torque ripple in a system. The proposed optimization is tested across four different load scenarios: 0 NM, 50 Nm, 100 Nm and 200 Nm. The TPCO method demonstrates better control of torque ripple at various load levels compared to the existing methods, offering improvements in performance, particularly by reducing the upper end of the torque ripple ranges in all cases.

**Table 3:** Comparison table of result parameters.

Optimization	Torque Ripple (Nm)	Difference	Torque Ripple (Nm)	Difference
	HHO (Abdel-Kader, 2023)		TPCO (Proposed)	
Case 1: Load: 0 Nm, Speed 1500	10:40	30	0:6	6
Case 2: Load: 50 Nm, Speed 2000	20:120	100	35:65	30
Case 3: Load: 100 Nm, Speed 2500	30:180	150	60:120	60
Case 4: Load: 200 Nm, Speed 3000	70:260	190	170:290	120

## Conclusion

In conclusion, this study introduces an enhanced Adaptive Sliding Mode Control (ASMC) strategy that significantly reduces torque ripple and improves speed control in Switched Reluctance Motor (SRM) drives. The proposed method integrates an outer-loop ASMC controller for speed regulation and an inner-loop ASMC controller with a hysteresis controller for torque control, ensuring better performance compared to traditional methods. The results show notable improvements in torque ripple reduction across all cases. Specifically, in **Case 1** (load: 0 Nm, speed: 1500), the torque ripple was reduced by **80%**, in **Case 2** (load: 50 Nm, speed: 2000), the reduction was **70%**, in **Case 3** (load: 100 Nm, speed: 2500), the improvement was **60%**, and in **Case 4** (load: 200 Nm, speed: 3000), the reduction was **36.84%**. These results demonstrate the effectiveness of the proposed method in minimizing torque ripple while maintaining accurate motor speed control. The enhanced ASMC strategy improves system stability, efficiency, and overall performance, making it a promising solution for SRM drive systems. This approach could lead to further advancements in motor control technologies and expand its applications in various industries. Further research will aim to expand the application to multi-motor propulsion systems and validate the approach through real-time implementation on a physical SRM drive setup.

## Data Availability

All data generated or analysed during this study is included in the main content of this publication.
